# Integrative Analysis of Transcriptome and Metabolome Reveals Regulatory Networks Associated with Flavonoids in Leaves of *Rhododendron hainanense* Under High-Temperature Stress

**DOI:** 10.3390/plants15060964

**Published:** 2026-03-20

**Authors:** Minghui Zhai, Enbo Wang, Jiaxuan Shi, Wendi Deng, Chengming Yan, Jian Wang, Xiqiang Song, Youhai Shi, Ying Zhao

**Affiliations:** 1 Key Laboratory of Ministry of Education for Genetics and Germplasm Innovation of Tropical Special Treesand Ornamental Plants, Hainan Biological Key Laboratory for Germplasm Resources of Tropical Special Ornamental Plants, College of Tropical Agriculture and Forestry, Hainan University, Haikou 570228, China; 23210907000001@hainanu.edu.cn (M.Z.); baroque2014@outlook.com (E.W.); 23220954000046@hainanu.edu.cn (J.S.); dwdwgl@hainanu.edu.cn (W.D.); ycm628@126.com (C.Y.); wjhnau@163.com (J.W.); songstrong@hainanu.edu.cn (X.S.); shiyouhai@163.com (Y.S.); 2 Hainan Institute of National Park, Haikou 570203, China

**Keywords:** *Rhododendron hainanense*, heat stress, transcriptomics, metabolomics, flavonoid biosynthesis

## Abstract

Heat stress severely impairs normal plant growth and yield, which significantly limits the horticultural and productive application of most *Rhododendron* species. In contrast, *Rhododendron hainanense* exhibits considerable heat tolerance due to its unique growing environment; however, the molecular mechanisms underlying its response to heat stress remain poorly understood. In this study, *R. hainanense* plants were subjected to heat stress treatment. Combined transcriptomic and metabolomic analyses identified 5454 differentially expressed genes and 152 differential metabolites. The results demonstrated that heat stress significantly induced the accumulation of flavonoids in *R. hainanense*. Notably, derivatives of myricetin, quercetin, and kaempferol were abundantly accumulated, suggesting their potential role in aiding plant defense against heat stress. The significant up-regulation of specific *Rh4CL* and *RhFLS* genes under high-temperature stress, coupled with the substantial accumulation of their flavonoid products (myricetin, quercetin, and kaempferol), indicates a potential role for these metabolites in the thermotolerance of *Rhododendron hainanense*. These findings provide novel insights into the heat stress response and flavonoid biosynthesis regulation in *R. hainanense*, highlighting the critical role of flavonoids in plant adaptation to heat stress. This study offers valuable references for the genetic improvement of *Rhododendron* cultivars with high stress resistance.

## 1. Introduction

Global climate warming and the increasing frequency of extreme heat events have established heat stress as a major abiotic threat affecting plant growth and development [1]. Extreme high-temperature events negatively impact various plant processes, including seed germination, photosynthesis, and water utilization, thereby disrupting normal growth and development. This stress reduces flowering rates and severely compromises both plant quality and yield, and in extreme cases, can lead to plant death. Such factors critically constrain the industrial development of heat-sensitive crops [2,3]. *Rhododendron hainanense* is an evergreen shrub belonging to the genus *Rhododendron* within the Ericaceae family. It is primarily distributed along low-altitude streams in the mountainous regions of southwestern China, such as Limu Mountain, Diaoluo Mountain, and Jianfengling in Hainan and Guangxi provinces [4]. The unique growth environment of *Rhododendron hainanense* endows it with greater heat tolerance compared to common rhododendron varieties. Most evergreen *Rhododendron* species are typically found in cool, humid high-altitude environments and are sensitive to high temperatures. Summer heat stress is a major factor limiting their growth and ornamental quality. When introduced to warmer regions, they often fail to acclimate to the high temperatures, suffering from physiological metabolic disorders and impeded photosynthesis, which leads to leaf yellowing, scorching, reduced plant vigor, and even death [5]. Therefore, investigating the regulatory mechanisms of plant responses to high-temperature stress and breeding heat-tolerant varieties is of great significance for enabling plants to cope with extreme heat events and promoting the industrial development of heat-sensitive crops.

Numerous studies have demonstrated that flavonoid content in plants is significantly correlated with stress tolerance, and increasing flavonoid accumulation can enhance plant resistance to adverse environmental conditions [6]. Flavonoids represent one of the largest classes of plant secondary metabolites, and their biosynthesis constitutes a widespread biochemical pathway in higher plants. The biosynthesis of flavonoids primarily involves the phenylpropanoid metabolism for precursor supply and the dedicated flavonoid biosynthesis pathway, which mainly involves the following enzymes: phenylalanine ammonia-lyase (PAL), cinnamate 4-hydroxylase (C4H), 4-coumarate:CoA ligase (4CL), chalcone synthase (CHS), chalcone isomerase (CHI), flavanone 3-hydroxylase (F3H), flavonoid 3′-hydroxylase (F3′H), flavonoid 3′,5′-hydroxylase (F3′5′H), flavonol synthase (FLS), dihydroflavonol 4-reductase (DFR), and anthocyanidin synthase (ANS/LDOX) [7].

Abiotic stresses induce the production, accumulation, and signaling of reactive oxygen species (ROS). Flavonoids, as secondary metabolites possessing antioxidant properties, play an effective role in scavenging ROS and preventing its overaccumulation, thereby aiding plants in better coping with environmental stresses [8,9,10,11,12]. Previous studies have shown a close association between flavonoid metabolism and plant responses to various abiotic stresses, including heat, drought, low temperature, and salinity. For instance, in rice, the *OsDUGT1* gene can glycosylate flavonoids, promoting their accumulation and the expression of heat-response-related genes, thereby conferring enhanced thermotolerance [13]. In roses, integrated transcriptomic and metabolomic data revealed that heat stress led to the significant enrichment of numerous differentially expressed genes and metabolites in the flavonoid biosynthesis pathway, indicating a crucial role for flavonoids in rose thermotolerance [14]. In *Scutellaria baicalensis*, *SbMYB8* was significantly upregulated under moderate drought stress, markedly increasing flavonoid synthesis and related gene expression [15]. In tea plants under drought stress, the expression of flavonoid biosynthesis-related genes, including *CHS*, *DFR*, *LAR*, and *ANS*, was downregulated during the early stages but upregulated as the stress prolonged [16,17]. Transgenic *Arabidopsis* overexpressing *AtUGT76E11*, which leads to excessive flavonoid accumulation, exhibited higher antioxidant capacity, reduced ROS accumulation, and enhanced salt stress tolerance [18]. *AtMYB111* in *Arabidopsis* is involved in regulating salt stress responses, and its suppression is significantly associated with reduced salt tolerance. Increased flavonoid content was linked to *AtMYB111* overexpression, suggesting flavonoid involvement in the *AtMYB111*-mediated salt stress tolerance mechanism [19]. Under drought and salt stress, the rice chalcone isomerase gene *OsCHI2* was up-regulated, and transgenic rice overexpressing *OsCHI2* exhibited enhanced photosynthetic activity under these stress conditions [20]. In rice, overexpression of *F3H* promoted the expression of salt- and heat-related genes, significantly increased the accumulation of kaempferol and quercetin, and improved tolerance to combined salt and heat stress [21].

Current research on *Rhododendron hainanense* has primarily focused on its physiological characteristics, as well as the discovery and functional validation of genes related to heat tolerance. However, few studies have investigated the regulatory role of flavonoids in response to high-temperature stress. In this study, we performed an integrated analysis of transcriptomic and metabolomic data to investigate the regulatory networks associated with flavonoids in response to high-temperature stress and to identify key genes that may play crucial roles by analyzing the expression patterns of flavonoid-related genes and the accumulation of flavonoid compounds under high-temperature conditions. This research explores the function of flavonoid components in *R. hainanense* under high-temperature stress, which can enrich the molecular theories of stress resistance in the field of molecular breeding and provide a basis for breeding rhododendron varieties with high flavonoid content and enhanced stress tolerance.

## 2. Results

### 2.1. Analysis of Metabolic Differences Between Heat Stress and Control Groups

**Figure 1:**
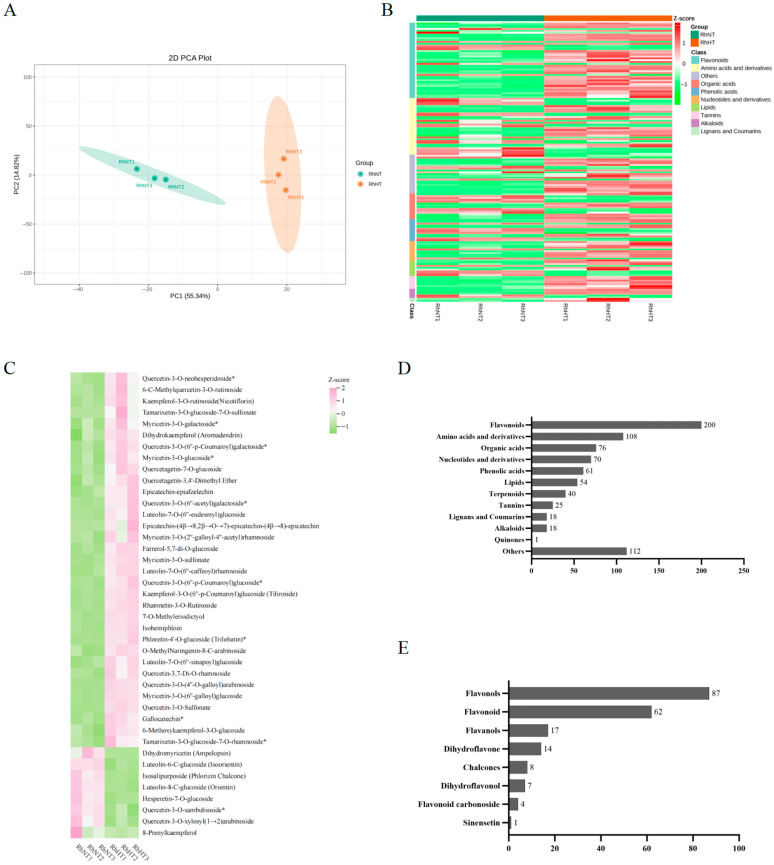
Metabolomic Profiling of *Rhododendron hainanense* under High-Temperature Stress. (**A**) Principal component analysis (PCA) of the metabolite expression dataset. PC1 and PC2 represent the first and second principal components, respectively. (**B**) Hierarchical clustering heatmap of differentially accumulated metabolites (DAMs) between the two treatment groups under high-temperature stress. The heatmap illustrates the expression patterns of metabolites across samples, with colors indicating relative metabolite abundance (red: upregulation; green: downregulation). (**C**) Heatmap analysis of flavonoid-related differentially accumulated metabolites. Colors represent relative metabolite abundance (pink: upregulation; green: downregulation). (**D**) Classification of all differentially accumulated metabolites, showing the categories and numbers of all DAMs identified. (**E**) Classification of flavonoid-related differentially accumulated metabolites, showing the categories and numbers of flavonoid DAMs identified.

To further investigate the impact of heat stress on the metabolite composition in *Rhododendron hainanense* leaves, the metabolite profiles of the heat-treated (HT) and control (CK) group leaves were identified using ultra-performance liquid chromatography-tandem mass spectrometry (UPLC-MS/MS). Principal component analysis (PCA) revealed significant differences in the metabolites of *R. hainanense* after heat treatment (Figure 1A). The orthogonal partial least squares-discriminant analysis (OPLS-DA) score plot revealed distinct metabolic profiles between treatment groups. The model parameters (R^2^X = 0.675, R^2^Y = 1, Q^2^ = 0.946) indicated a robust fit and high predictive ability, demonstrating that high-temperature stress induced substantial alterations in the metabolome of *Rhododendron hainanense*. A total of 783 metabolites were detected across the heat-stressed (HT) and control (CK) samples. Notably, flavonoids were the most abundant category, comprising 200 compounds (25.54% of the total) (Figure 1D).

Differentially accumulated metabolites (DAMs) between CK and HT samples were identified based on screening criteria of variable importance in projection (VIP) ≥ 1 from the OPLS-DA model and a fold change ≥ 2 or ≤0.5. This analysis yielded 152 DAMs, comprising 101 upregulated and 51 downregulated metabolites (Figure 1B). Among these, 41 were flavonoid compounds, representing 26.97% of all DAMs, with 33 upregulated and 8 downregulated (Figure 1C,E).

To gain deeper insight into the accumulation mechanism of flavonoids under heat stress, we focused on metabolites enriched in the flavonoid biosynthesis pathway. Based on KEGG analysis, 30 metabolites were enriched in the flavonoid biosynthesis pathway (ko00941), including 7 dihydroflavones, 5 dihydroflavonols, 5 flavanols, 4 chalcones, 4 flavones, 3 flavonols, and 2 phenolic acids. Among these 30 metabolites, 2 were significantly upregulated and 3 were downregulated. Furthermore, 21 metabolites were enriched in the flavone and flavonol biosynthesis pathway (ko00944), consisting of 15 flavonols and 6 flavones, with 2 significantly upregulated and 1 downregulated.

Following heat treatment, the contents of dihydrokaempferol (aromadendrin), gallocatechin, kaempferol-3-O-rutinoside (nicotiflorin), and quercetin-3-O-sulfate increased significantly, suggesting their potential key roles in the heat stress response of *R. hainanense*. Conversely, the contents of hesperetin-7-O-glucoside, dihydromyricetin (ampelopsin), and quercetin-3-O-sambubioside decreased significantly, which may be associated with the heat-induced suppression of relevant biosynthetic enzyme activities. Among the significantly altered flavonoid DAMs, derivatives of myricetin, quercetin, luteolin, and kaempferol were predominantly upregulated. This suggests that the biosynthesis of these specific flavonoids is activated during the heat stress response, potentially aiding the plant in mitigating heat-induced damage.

### 2.2. Analysis of Transcriptomic Differences Between Heat Stress and Control Groups

To further investigate the molecular mechanisms underlying flavonoid biosynthesis in *R. hainanense* under heat stress, transcriptome sequencing was performed on leaves from the HT and CK groups to analyze differential gene expression. A total of 294,816,746 raw reads were generated from the six samples. After quality control, 288,605,690 clean reads were obtained, amounting to 43.29 Gb of clean data. All samples exhibited an error rate of 0.03%. The percentages of Q20 and Q30 bases ranged from 97.81% to 97.93% and from 93.56% to 93.84%, respectively, indicating high-quality data suitable for subsequent bioinformatic analysis.

**Figure 2:**
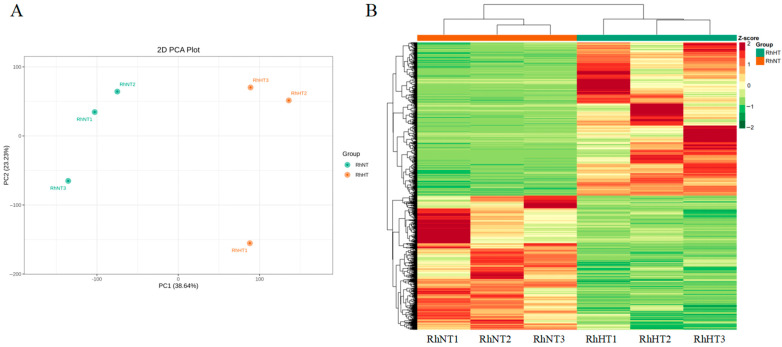
Transcriptomic changes in *Rhododendron hainanense* under heat stress. (**A**) Principal component analysis (PCA) of all gene transcripts after heat treatment. PC1 and PC2 represent the first and second principal components, respectively. (**B**) Heatmap of Gene Expression Clustering for All Transcripts under High-Temperature Stress. The red bar at the top of the heatmap indicates the three NT (control) samples, while the green bar indicates the three HT (high-temperature treatment) samples. Colors represent the relative expression levels of genes, with red indicating upregulation and green indicating downregulation.

The transcriptome data were aligned to the *R. hainanense* genome, with alignment rates ranging between 86.53% and 91.18%. A total of 35,543 genes were identified. Principal component analysis (PCA) revealed clear separation between samples after heat stress, while samples within the same group clustered closely, demonstrating good reproducibility (Figure 2A). Cluster heatmap analysis showed distinct gene expression patterns in *R. hainanense* following heat treatment (Figure 2B). Applying the thresholds of |log_2_(fold change)| ≥ 1 and a false discovery rate (FDR) < 0.05, a total of 5454 differentially expressed genes (DEGs) were identified, comprising 2911 upregulated and 2543 downregulated genes.

### 2.3. GO and KEGG Enrichment Analyses of Heat Stress and Control Groups

**Figure 3:**
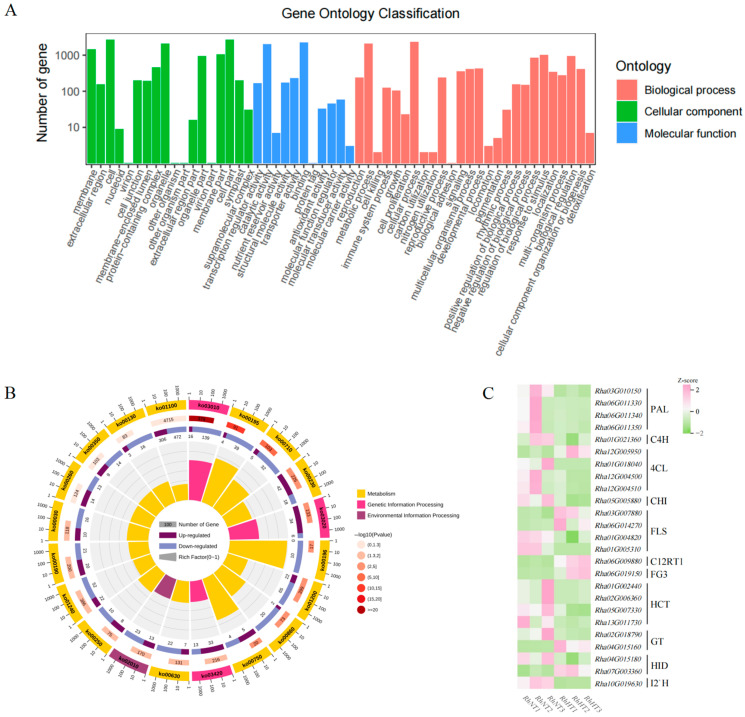
GO Enrichment Analysis, KEGG Enrichment Analysis, and Expression Heatmap of Flavonoid Biosynthesis-Related Genes in *Rhododendron hainanense* under High-Temperature Stress. (**A**) Classification statistics of GO enrichment annotations. (**B**) Chord diagram of KEGG enrichment analysis. (**C**) Expression heatmap of candidate structural genes involved in flavonoid biosynthesis.

To better understand the functions and roles of the differentially expressed genes (DEGs), we annotated them using the Gene Ontology (GO) and Kyoto Encyclopedia of Genes and Genomes (KEGG) databases. Among the 5454 DEGs annotated to GO terms, they were categorized into 55 functional groups across three major ontologies. In the “Cellular Component” ontology, 3088 genes were annotated, with “cell”, “organelle”, and “cell part” being the most enriched terms. Within “Molecular Function”, 3110 genes were annotated, predominantly enriched in terms such as “binding”, “catalytic activity”, and “transporter activity”. For “Biological Process”, 2849 genes were annotated, with “metabolic process”, “cellular process”, and “response to stimulus” showing the highest enrichment (Figure 3A). Notably, within “Biological Process”, 22 DEGs were associated with the flavonoid biosynthetic pathway. In the “Molecular Function” category, 15 DEGs were linked to quercetin 3-O-glucosyltransferase and quercetin 7-O-glucosyltransferase activity, while 2 DEGs were associated with naringenin 3-dioxygenase activity and 2 with 2-hydroxyisoflavone dehydratase activity.

To further elucidate the biosynthetic processes and metabolic pathways of flavonoids, the DEGs were annotated against the KEGG database. KEGG analysis revealed that 1773 DEGs were annotated to 134 pathways across 5 major categories. Among these, the ribosome, photosynthesis, and carbon fixation pathways were the most significantly enriched. In terms of the number of enriched genes, metabolic pathways, biosynthesis of secondary metabolites, and plant–pathogen interaction were the most prominent (Figure 3B). Based on previous evidence linking flavonoids to plant stress responses and our annotation results, we focused on flavonoid-related pathways. A total of 25 genes involved in flavonoid biosynthesis were identified from four key pathways: Phenylpropanoid biosynthesis, Flavonoid biosynthesis, Isoflavonoid biosynthesis, and Flavone and flavonol biosynthesis. Heatmap cluster analysis demonstrated that the expression of these genes was significantly up- or downregulated following heat treatment (Figure 3C).

### 2.4. Flavonoid Biosynthetic Pathways After Heat Stress Treatment

**Figure 4:**
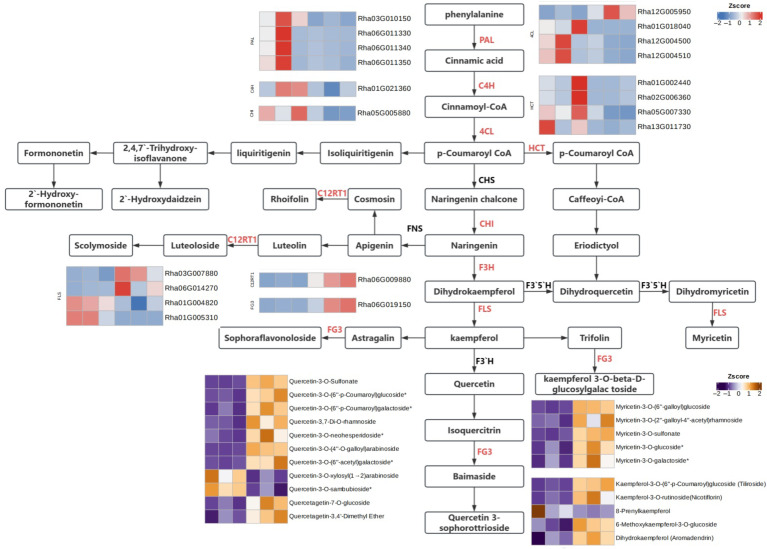
Regulatory network of flavonoid biosynthesis and expression profiles of key genes and metabolites. Genes shown in red text in the figure represent those that were identified as differentially expressed in this study. Color gradients from blue to red represent low to high gene expression levels, respectively. Gradients from purple to yellow indicate low to high metabolite accumulation levels, respectively.

Based on KEGG pathway annotation results, the flavonoid biosynthetic mechanism was constructed by integrating differential genes encoding key biosynthetic enzymes and the corresponding differentially accumulated metabolites (Figure 4). Following heat stress treatment, the expression of genes related to *RhPAL* and *RhC4H* was downregulated; however, the levels of their catalytic products, cinnamic acid and coumaric acid (precursors for flavonoid synthesis), showed no significant change. Among the *Rh4CL* genes, one was upregulated while three were downregulated, yet the level of the key intermediate p-coumaroyl-CoA remained largely unaltered. Similarly, the expression of genes associated with the key enzyme *RhCHI* decreased, but the accumulation of its catalytic product, naringenin, did not change significantly. The expression of *RhHCT* genes was reduced, while the content of its product, dihydroquercetin, exhibited minimal variation.

For *RhFLS*, two genes were upregulated and two were downregulated. The levels of its direct products, kaempferol, myricetin, and quercetin, remained stable, whereas the contents of their respective derivatives increased significantly. This discrepancy in expression patterns may be attributed to the involvement of related genes in different branch pathways. The downregulated expression of *RhFG3* genes corresponded with a decreased accumulation of some quercetin derivatives. The upregulated expression of *RhC12RT1* led to increased accumulation of kaempferol-3-O-rutinoside. The diverse expression patterns of *RhGT1* genes were associated with an increased accumulation of some anthocyanin-related metabolites.

Under heat stress, the expression of several key genes in flavonoid biosynthesis increased. Correspondingly, the accumulation of many flavonoid metabolites, particularly derivatives of kaempferol, myricetin, and quercetin, was significantly enhanced. Among these, the following upregulated genes were identified: *Rh4CL* (Accession: XM_058372331; Gene ID: 131336474), two *RhFLS* genes (Accession: XM_058351284, Gene ID: 131320559; Accession: XM_058341585, Gene ID: 131313328), and *RhC12RT1* (Accession: XM_058348998; Gene ID: 131318942). Further exploration of the interactions between these genes and their corresponding metabolites will contribute to a better understanding of their functions and regulatory mechanisms in stress responses.

### 2.5. Analysis of Transcription Factor Families in Heat Stress and Control Groups

**Figure 5:**
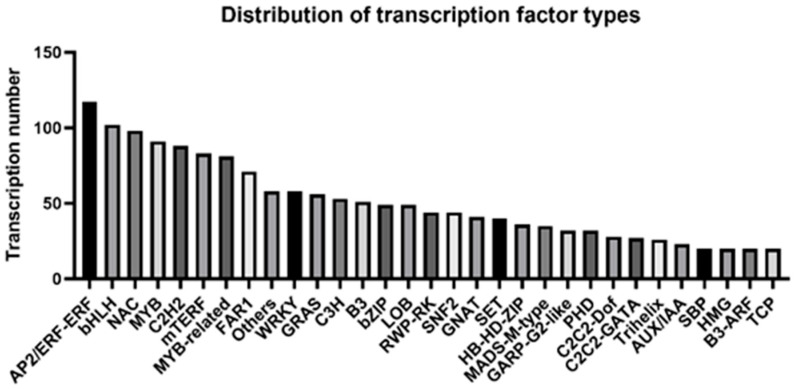
Number of transcription factors (Top 30) in *R. hainanense*.

Transcription factors (TFs) act as master regulators of gene expression and play crucial roles in plant growth, development, and responses to environmental stresses, including the regulation of metabolic genes. Through transcriptome analysis, TFs in the samples were identified and classified. A total of 2035 TFs were annotated into 94 families, with the top 30 most abundant families displayed (Figure 5). The results showed significant enrichment of stress-associated TF families in *Rhododendron hainanense*, including AP2/ERF-ERF, bHLH, NAC, MYB, WRKY, and bZIP.

After heat stress treatment, 230 differentially expressed TFs belonging to 61 families were identified (Supplementary Table S1). Families such as AP2/ERF-ERF, bHLH, NAC, MYB-related, MYB, FAR1, and GRAS contained a higher number of TFs. Specifically, 25 differentially expressed genes were annotated as AP2/ERF-ERF, 11 as NAC, and 11 as MYB-related. Notably, NAC transcription factors may act as upstream master regulators, modulating the expression of downstream MYB or AP2/ERF genes, which in turn precisely control flavonoid biosynthetic genes. This regulatory cascade potentially enhances flavonoid accumulation, aiding the plant in coping with stress. In addition to the transcription factor families discussed above, we identified 4 differentially expressed heat shock factor (HSF) genes under high-temperature stress, including members of the HSFA and HSFB subfamilies. These HSFs are known to function as master regulators of heat stress responses by activating downstream heat shock protein genes and other stress-responsive targets. Under heat stress, these TFs are activated and likely play important roles in the heat stress response and secondary metabolite biosynthesis in *R. hainanense*. Further investigation is warranted to elucidate the detailed regulatory mechanisms governed by these TFs.

### 2.6. Metabolite-Gene Correlation and Weighted Gene Co-Expression Network Analysis

**Figure 6:**
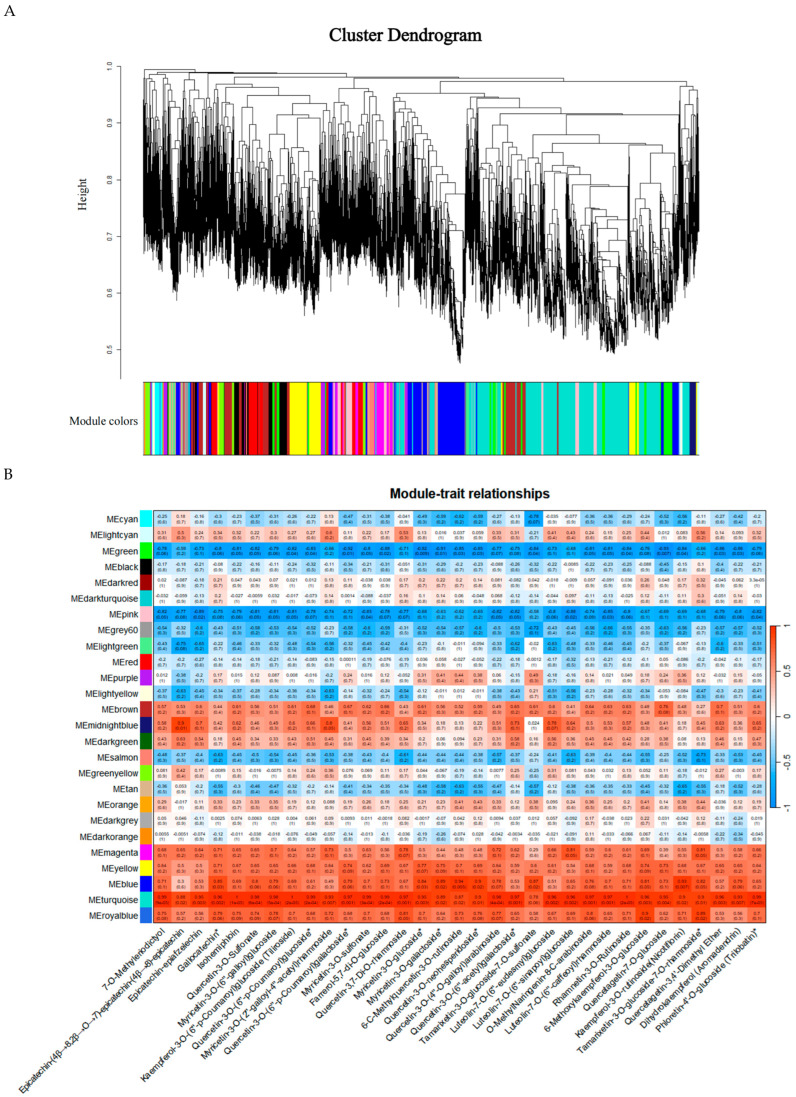

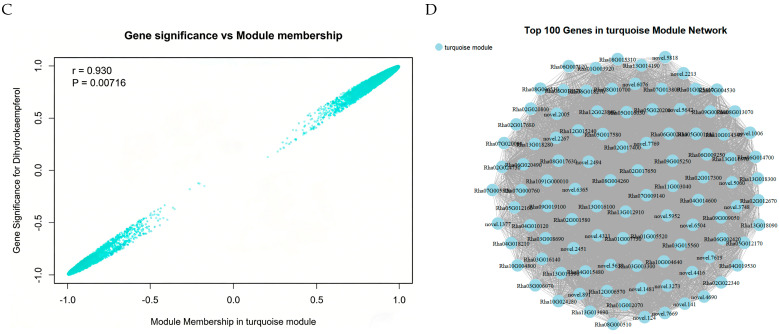
Weighted Gene Co-expression Network Analysis (WGCNA) of *Rhododendron hainanense* under Heat Stress. (**A**) Hierarchical clustering dendrogram. (**B**) Heatmap of module-sample correlations (red indicates positive correlation, blue indicates negative correlation). (**C**) Scatter plot of Gene Significance (GS) versus Module Membership (MM). MM represents the correlation between a gene and the module eigengene, indicating the degree to which a gene belongs to the module. GS represents the correlation between gene expression and a specific trait (here, Dihydrokaempferol). Genes located in the upper right quadrant are typically considered functional hub genes. (**D**) Co-expression network of the top 100 hub genes identified in the turquoise module.

To further elucidate the biosynthetic mechanisms of flavonoids and the molecular mechanisms underlying the heat stress response, Weighted Gene Co-expression Network Analysis (WGCNA) was performed based on integrated transcriptomic and metabolomic data. Using 34,067 genes obtained after filtering low-expression genes (FPKM < 0.5), a gene co-expression network was constructed, identifying 26 distinct co-expression modules. Association analysis was conducted between these gene modules and 33 significantly upregulated flavonoid metabolites used as phenotypic traits. The results indicated that the turquoise module exhibited a significant positive correlation with the upregulated flavonoids, showing correlation coefficients above 0.8 for most metabolites (Figure 6A,B). The highest correlation was observed with Rhamnetin-3-O-Rutinoside. In contrast, the green module displayed a significant negative correlation with these flavonoids, with most correlation coefficients less than −0.7.

Analysis of the turquoise module revealed that two-thirds of the differentially expressed genes (DEGs) were enriched within it, suggesting that genes in this module play a crucial role in the plant’s response to high-temperature environments. Furthermore, this module contained multiple key genes encoding flavonoid biosynthetic enzymes, including *RhPAL*, *Rh4CL*, *RhC4H*, *RhCHI*, *RhHCT*, *RhFLS*, *RhF3′H*, *RhF3′5′H*, *Rh5GT*, *RhHID*, *RhI2′H*, *RhC12RT1*, and *RhFG3*. Furthermore, analysis of genes in the turquoise module using dihydrokaempferol as a trait revealed that *RhHCT*, a gene involved in flavonoid biosynthesis, was identified as a hub gene in this module, suggesting that it may play an important role in the accumulation of dihydrokaempferol (Figure 6C,D). However, further functional validation of this gene is required. The sequence data for the gene (Accession number: XM_05838216.1; Gene ID: 131310938) were obtained from the NCBI database.

### 2.7. qRT-PCR Validation of Transcriptomic Data

**Figure 7:**
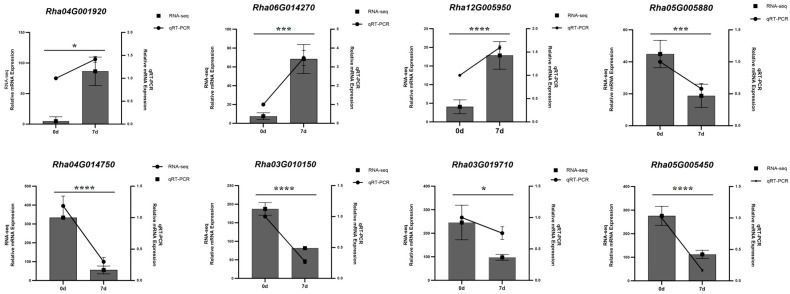
Expression patterns of eight flavonoid biosynthesis-related genes under heat stress as detected by qRT-PCR. The left vertical axis coordinate represents FPKM from RNA-seq; the right vertical axis coordinate represents the relative gene expression level from qRT-PCR. Error bars represent standard deviation (SD). Different asterisks in the figure indicate statistically significant differences (*p* ≤ 0.05).

To further validate the reliability of the RNA-Seq data, quantitative real-time PCR (qRT-PCR) was performed. Eight genes involved in the flavonoid biosynthesis pathway were selected for verification. The results demonstrated that the expression patterns of these eight genes were consistent with the trends observed in the RNA-Seq dataset (Figure 7), confirming the reliability of the transcriptomic data obtained in this study.

## 3. Discussion

Flavonoids, as antioxidants, play a crucial role in plant adaptation to environmental stresses, including high temperature, by helping plants cope with adverse conditions [22]. Through integrative multi-omics analysis, this study analyzed the accumulation patterns of flavonoids in *Rhododendron hainanense* under high-temperature stress and provided potential insights into their molecular biosynthetic networks. Metabolomic analysis indicated that flavonols (e.g., kaempferol, quercetin, and myricetin derivatives) may be key metabolites involved in the response of *R. hainanense* to heat stress. Consistent with our findings, the rice heat-tolerant mutant *rel1-D* exhibited enhanced heat tolerance through upregulation of key flavonoid biosynthesis genes and subsequent accumulation of flavonoid metabolites [23]. Similarly, the banana heat shock transcription factor *MaHSF11* acts as a key regulatory switch by directly activating flavonol synthase genes to promote the synthesis of flavonols such as myricetin, thereby assisting in heat stress adaptation [24]. These findings suggest that flavonoid-mediated thermotolerance mechanisms may be conserved across plant species, with these compounds protecting cells from oxidative damage by scavenging excess reactive oxygen species (ROS), thereby enhancing plant heat tolerance [25]. Notably, in this study, flavonoid-related differential metabolites accounted for 26.97% of all differential metabolites, with over 80% of them showing upregulated accumulation—a proportion substantially higher than that of other metabolite categories. This further suggests that flavonoid metabolism may play a particularly important role in the heat adaptation of *R. hainanense*.

To elucidate the regulatory network underlying flavonoid-mediated responses to high-temperature stress in Rhododendron hainanense, transcriptomic analysis was performed. The results revealed that the expression levels of most flavonoid biosynthesis-related genes, including *RhPAL*, *Rh4CL*, *RhC4H*, *RhHCT*, *RhCHI*, *RhCYP73A*, and *RhCYP98A*, were downregulated following high-temperature stress. This finding is consistent with previous observations in Oriental hybrid lilies, where high-temperature stress downregulated anthocyanin biosynthesis pathway genes, leading to reduced anthocyanin accumulation [26]. However, the accumulation levels of downstream metabolites, including naringenin chalcone, naringenin, naringin, hesperetin, apigenin, and vitexin, showed no significant differences between treatment groups. This discrepancy may be explained by a dynamic shift in the rate-limiting steps of the flavonoid biosynthesis pathway in response to environmental conditions. Under control conditions, upstream enzymes such as PAL or CHS may serve as the primary rate-limiting steps. However, under high-temperature stress, plants may shift the rate-limiting step to downstream modifying enzymes such as FLS or glycosyltransferases through post-transcriptional regulation or protein modification [27]. In this scenario, the limiting effect of downregulated upstream gene expression on overall metabolic flux is attenuated; as long as the activity of existing enzymes is sufficient to maintain intermediate metabolite supply, the levels of key node metabolites can remain stable.

Alternatively, molecular chaperones such as heat shock proteins (HSPs), which are abundantly expressed under high-temperature stress, may help maintain the proper conformation and catalytic activity of existing enzymes, including PAL, CHS, and CHI [28]. Thus, despite reduced mRNA synthesis, the degradation of existing proteins may be delayed and their activity sustained, ensuring the continued production of node metabolites. Flavonol synthase (FLS) is widely recognized as a key enzyme in flavonoid and flavonol biosynthesis, catalyzing the formation of kaempferol and myricetin derivatives, and has been suggested to play a role in plant stress tolerance [29]. In the present study, *RhFLS* genes exhibited diverse expression patterns, with two genes upregulated and two downregulated under high-temperature stress. Concomitantly, the accumulation levels of their metabolic products, including kaempferol and quercetin derivatives, were significantly upregulated. FLS is considered to occupy a key branch point in the flavonoid metabolic pathway, potentially competing with the anthocyanin biosynthesis pathway for the common substrate dihydroflavonols. Overexpression of rice *OsFLS* in transgenic tobacco resulted in significantly increased levels of kaempferol-3-O-rutinoside in petals, accompanied by markedly reduced anthocyanin levels [30]. Similarly, overexpression of *DoFLS1* from Dendrobium officinale in Arabidopsis significantly suppressed the expression of anthocyanin biosynthesis structural genes, including CHS, CHI, DFR, ANS, and UFGT [29]. Based on these findings, we propose that the downregulated *RhFLS* genes identified in this study may be associated with anthocyanin biosynthesis or other branching pathways, while the upregulated *RhFLS* genes, given their higher expression levels under high-temperature stress, might competitively utilize dihydroflavonol substrates, potentially leading to a redirection of metabolic flux toward flavonol biosynthesis. This hypothesized shift could contribute to the accumulation of thermotolerance-related metabolites and may be involved in the heat stress response of *Rhododendron hainanense*. However, further functional validation is needed to confirm these inferred relationships.

Transcription factors (TFs) serve as central hubs in regulatory networks, playing a pivotal role in integrating environmental signals and activating downstream target genes. In this study, we identified 230 differentially expressed transcription factors belonging to 61 families, constituting a complex regulatory hierarchy (Figure 5). Among these, the AP2/ERF family contained the highest number of differentially expressed genes (25 members), suggesting that this family may play a prominent role in the response of *Rhododendron hainanense* to high-temperature stress. AP2/ERF transcription factors have been extensively documented as key regulators in plant responses to various abiotic stresses, including high temperature, drought, and salinity. These factors typically function by directly binding to GCC-box or DRE elements in the promoters of downstream target genes, thereby activating stress-responsive gene expression [31]. MYB transcription factors, particularly those of the R2R3-MYB type, represent the most well-characterized regulators of flavonoid metabolism. They frequently form MBW complexes with bHLH and WD40 proteins to specifically activate distinct branches of the flavonoid biosynthetic pathway [32]. In this study, we identified 11 differentially expressed MYB and MYB-related transcription factors, among which may be key regulators of flavonol biosynthesis. For instance, *AtMYB12* in Arabidopsis has been demonstrated to directly activate FLS and CHS expression, promoting flavonol accumulation [33]. NAC transcription factors also featured prominently, with 11 differentially expressed members identified in this study. NAC family members commonly function as upstream regulators involved in growth and development, senescence, and stress responses [34]. In chrysanthemum, CmVNI2 has been shown to directly bind the promoters of *CmF3H* and *CmMYB3*, regulating flavonol biosynthesis under low-temperature conditions [35]. This raises the possibility that the differentially expressed NAC transcription factors identified in *R. hainanense* may serve as intermediaries that transmit upstream heat stress signals to downstream flavonoid biosynthesis modules. Based on these observations, we propose a hierarchical regulatory model in which differentially expressed AP2/ERF members in *R. hainanense* occupy the apex of the regulatory network. These factors may function both by directly activating certain flavonoid biosynthetic genes (such as the upregulated *RhFLS* genes) and by modulating downstream MYB or NAC transcription factors, thereby generating a cascade amplification effect that precisely coordinates stress responses with secondary metabolism.

Through integrated transcriptomic and metabolomic analysis, this study identified key flavonoids (including kaempferol, quercetin, and myricetin derivatives) and candidate genes (*Rh4CL* and *RhFLS*) that may play critical roles in the response of Rhododendron hainanense to high-temperature stress. These findings offer novel insights with potential applications for the sustainable development of the rhododendron industry and provide a scientific basis for production practices in the context of global warming. The Rh4CL and RhFLS genes, identified as being closely associated with flavonoid accumulation and heat tolerance in this study, could serve as direct targets for marker-assisted selection (MAS) in breeding programs following validation through physiological assays and functional characterization. Furthermore, the accumulation levels of specific flavonols (e.g., quercetin-3-O-rutinoside and kaempferol-3-O-rutinoside) in leaves under high-temperature stress may serve as early biochemical indicators for assessing thermotolerance in plant germplasm. Such indicators could enable preliminary screening of breeding materials for heat tolerance, thereby significantly shortening breeding cycles. Additionally, our findings may inform precision management practices in nurseries. For instance, controlled stress treatments applied prior to the onset of high-temperature stress could promote the pre-accumulation of protective flavonoids, enhancing plant tolerance during subsequent heat stress events. This approach could mitigate heat damage symptoms and ensure seedling quality and survival rates.

While this study has elucidated the regulatory networks associated with flavonoid-mediated responses to high-temperature stress in *Rhododendron hainanense*, several limitations should be acknowledged and addressed in future investigations. First, the experimental conditions present certain constraints. This study was conducted in controlled environment chambers, which, despite enabling variable control, may not fully replicate the complex conditions of field environments. Notably, the high-temperature treatment (40 °C/30 °C) was inevitably accompanied by a significant increase in vapor pressure deficit (VPD). Plant stomatal responses and water status under high-temperature conditions are, in fact, co-determined by both temperature and VPD [36]. The present study could not completely disentangle the confounding effects of high temperature and elevated VPD. Therefore, extrapolation of these findings to field conditions warrants caution. Future experiments incorporating varying light intensities and humidity gradients will be necessary to dissect the interactive effects of light, temperature, and water factors. Second, this study lacks supporting physiological and phenotypic data. Key parameters under high-temperature stress, such as photosynthetic performance (e.g., net photosynthetic rate, Pn; maximum photochemical efficiency, Fv/Fm), reactive oxygen species accumulation levels (e.g., histochemical staining and quantification of H_2_O_2_ and O_2_^−^), and membrane lipid peroxidation (e.g., malondialdehyde content), were not measured. This limits our ability to establish direct causal relationships between flavonoid accumulation and physiological improvements, such as protection of the photosynthetic apparatus and mitigation of oxidative damage. Third, the key candidate genes and regulatory models proposed in this study are currently based on correlational analysis and lack functional validation. Future studies employing heterologous overexpression of candidate genes in Arabidopsis or other model systems will be essential to validate their functions and further elucidate the underlying regulatory mechanisms.

## 4. Materials and Methods

### 4.1. Plant Materials

The *Rhododendron hainanense* plants used in this experiment were cultivated at the Agricultural Science Base of Hainan University, Danzhou Campus (19°30′ N, 109°29′ E). The plants were grown in a substrate mixture consisting of peat soil, Kanuma soil, and Akadama soil in a 3:1:1 ratio. Ten 5-year-old seedlings with consistent growth status were selected and subjected to treatments in an artificial climate chamber, where temperature and light conditions were uniformly maintained for all plants.

To ensure uniform initial growth conditions, the selected seedlings were first acclimated in the climate chamber at 25 °C/22 °C with a photoperiod of 14 h/10 h (light/dark), light intensity of 2000 lx, and relative humidity of 70% for 7 days. Subsequently, the 10 seedlings were evenly divided into two groups and exposed to different temperature regimes for 7 days. The high-temperature treatment group (HT) was maintained at 40 °C/30 °C with 14 h/10 h photoperiod, 2000 lx light intensity, and 70% relative humidity, while the control group (CK) was maintained at 25 °C/22 °C under otherwise identical conditions. Three plants per treatment group were used as biological replicates for sample collection. On the 7th day of treatment, at 10:00 a.m., leaf samples (the 3rd to 5th whorl leaves) were collected from both the HT and CK groups, with all samples taken from the same leaf position. The collected samples were immediately frozen in liquid nitrogen and stored at −80 °C until further analysis. For both transcriptome and metabolome analysis, three biological replicates were collected per treatment, with each replicate consisting of samples taken from an individual plant.

### 4.2. Metabolite Identification and Quantification

Metabolite profiling was conducted using ultra-performance liquid chromatography coupled with tandem mass spectrometry (UPLC-MS/MS). Leaf samples from both the HT and CK groups were vacuum freeze-dried and ground into a fine powder at 30 Hz for 1.5 min. Subsequently, 100 mg of the powder was dissolved in 1.2 mL of 70% methanol extraction solvent, vortexed thoroughly, and stored overnight at 4 °C. Following incubation, the samples were centrifuged at 12,000 rpm for 10 min. The supernatant was then collected, filtered through a 0.22 μm pore-size microporous membrane, and stored for subsequent UPLC-MS/MS analysis.

The data acquisition system primarily consisted of a UPLC unit (UPLC, SHIMADZU Nexera X2, www.shimadzu.com.cn/, accessed on 6 April 2021) and a tandem mass spectrometer (MS, Applied Biosystems 6500 Q TRAP, www.appliedbiosystems.com.cn/, accessed on 6 April 2021). Chromatographic separation was performed on an Agilent SB-C18 column (1.8 µm, 2.1 mm * 100 mm) maintained at 40 °C. The mobile phase comprised solvent A (ultrapure water with 0.1% formic acid) and solvent B (acetonitrile with 0.1% formic acid). The elution gradient was programmed as follows: 5% B at 0 min, linearly increasing to 95% B over 9.00 min, holding at 95% B for 1 min (9.00–10.00 min), decreasing to 5% B from 10.00 to 11.10 min, and finally equilibrating at 5% B until 14.00 min. The flow rate was set at 0.35 mL/min, and the injection volume was 2 µL.

Mass spectrometric data were acquired using an AB Sciex QTRAP^®^ 6500+ triple quadrupole-linear ion trap mass spectrometer equipped with an ESI-Turbo ion spray interface, controlled by Analyst 1.6.3 software. The ESI source operating parameters were as follows: ion source, turbo spray; source temperature, 550 °C; ion spray voltage (IS), 5500 V (positive ion mode) or −4500 V (negative ion mode); ion source gas I (GSI), gas II (GSII), and curtain gas (CUR) were set at 50, 60, and 25.0 psi, respectively; and the collision-induced dissociation parameters were set to high.

Metabolite identification in this study was performed based on secondary mass spectrometry information, using chromatographic grade standards (BioBioPha/Sigma-Aldrich) to construct the MWDB database. According to the metabolomics standards initiative (MSI) identification levels, the identified metabolites in this study were classified as MSI level 1 (confirmed by matching retention time (Rt), precursor ion (Q1), product ion (Q3), declustering potential (DP), and collision energy (CE)) and MSI level 2 (putatively identified based on MS/MS spectral matching with database reference spectra). Relative quantification was performed using multiple reaction monitoring (MRM) mode, with metabolites detected using five parameters (declustering potential, collision energy, retention time, precursor ion *m*/*z*, and product ion *m*/*z*) to determine relative metabolite abundances across samples, thereby obtaining qualitative and quantitative data.

To evaluate data quality, principal component analysis (PCA) and orthogonal partial least squares-discriminant analysis (OPLS-DA) were performed on the samples. Differential metabolites were identified by combining fold change analysis with variable importance in projection (VIP) values from the OPLS-DA model. The screening criteria were as follows: (1) metabolites with a fold change ≥ 2 or ≤0.5 were selected; (2) metabolites with VIP ≥ 1 were selected. Hierarchical cluster analysis was performed using R 3.5.1 software.

### 4.3. Transcriptome Sequencing, Assembly, and Functional Annotation

Total RNA was extracted from *Rhododendron hainanense* leaves using a Total RNA Extraction Kit (TIANGEN, Beijing, China). The integrity of the extracted RNA and the absence of DNA contamination were assessed via agarose gel electrophoresis. RNA concentration was precisely measured using a Qubit 2.0 Fluorometer (Life Technologies, CA, USA), and RNA integrity was accurately evaluated with an Agilent 2100 Bioanalyzer (Agilent Technologies, CA, USA). Strand-specific libraries were constructed using the NEBNext^®^ Ultra™ RNA Library Prep Kit for Illumina (NEB, Ipswich, MA, USA) following the manufacturer’s instructions. Library concentration was initially quantified using a Qubit 2.0 fluorometer, and the insert size was assessed using an Agilent 2100 Bioanalyzer. Subsequently, the libraries were sequenced on the Illumina HiSeq platform, generating 150 bp paired-end reads.

Raw sequencing reads were processed to obtain clean data by: 1 removing reads containing adapter sequences; 2 discarding paired-end reads if either read contained more than 10% unknown nucleotides (N); and 3 filtering out paired-end reads where over 50% of the bases in either read had a quality score (Q) ≤ 20. Clean reads were aligned to the *Rhododendron hainanense* reference genome (sequenced by BGI, Shenzhen, China) using HISAT2. Gene expression levels were quantified using featureCounts v1.6.2 and normalized to fragments per kilobase of transcript per million mapped reads (FPKM). The FPKM values were used exclusively for expression visualization. Raw read counts were employed for DESeq2 analysis, and differential expression analysis was performed using the DESeq2 R package. Genes with an adjusted absolute |log_2_Fold Change| ≥ 1 and a false discovery rate (FDR) < 0.05 were considered significantly differentially expressed. Gene Ontology (GO) enrichment and Kyoto Encyclopedia of Genes and Genomes (KEGG) pathway enrichment analyses for the differentially expressed genes (DEGs) were performed based on the hypergeometric test. Functional annotation of genes was carried out using the KEGG, GO, and Clusters of Orthologous Groups (KOG) databases.

To construct co-expression regulatory networks between genes and flavonoids, weighted gene co-expression network analysis (WGCNA) was performed using the R package (version 3.5.1), with significantly up-regulated flavonoids under high-temperature stress serving as the phenotypic traits [37]. First, based on the selection criterion for the soft-thresholding power (β), β = 16 was chosen as the optimal soft-thresholding power to ensure a scale-free topology fit index (R^2^) greater than 0.8. Subsequently, hierarchical clustering was performed based on the dissimilarity coefficient (1-TOM) among genes, and gene modules were identified using the dynamic tree-cutting method. The main parameters were set as follows: minimum module size (minModuleSize) = 30, and the threshold for merging similar modules (mergeCutHeight) = 0.25. The module eigengene (ME), representing the overall expression pattern of each module, was calculated. Pearson correlation analysis was performed between MEs and the content of various flavonoids to determine correlation coefficients and significance (*p*-values), and a module-trait correlation heatmap was generated. To identify hub genes in key modules, the following metrics were calculated: Gene Significance (GS): the absolute value of the correlation between gene expression and the target flavonoid trait. A GS > 0.2 was set as the screening threshold. Module Membership (MM): the correlation between gene expression and the module eigengene. |MM| > 0.8 was set as the screening threshold. Intramodular connectivity (kWithin): the connection strength between a given gene and other genes within the same module. Genes ranking within the top 10–20% of intramodular connectivity were selected. Genes simultaneously satisfying |MM| > 0.8, GS > 0.2, and ranking within the top 20 in terms of kWithin within the module were defined as candidate hub genes. For co-expression analysis, the top 100 hub genes extracted from key modules, together with flavonoid biosynthesis structural genes, were imported into Cytoscape software (version 3.10.3) for network visualization.

### 4.4. RT-qPCR Validation

Eight genes associated with flavonoid biosynthesis were selected for validation by real-time quantitative PCR (RT-qPCR). Total RNA was extracted from *Rhododendron hainanense* leaves using a Total RNA Extraction Kit (TIANGEN, Beijing, China). The extracted RNA was then reverse-transcribed into cDNA using a reverse transcription kit from Vazyme (Nanjing, China). Gene-specific primers were designed using Primer Premier 5.0. RT-qPCR was performed on a real-time PCR system (Bio-system, Roanoke, VA, USA). The 18S rRNA gene was used as the internal reference, and the relative expression levels of the target genes were calculated using the 2^−ΔΔCt^ method.

## 5. Conclusions

High-temperature stress severely negatively impacts normal plant growth and development, but to a certain extent, it can also induce the biosynthesis of flavonoids. In this study, *Rhododendron hainanense* was subjected to high-temperature stress, and an integrated analysis of metabolomic and transcriptomic data was performed to preliminarily investigate the flavonoid biosynthesis mechanism under high-temperature conditions. A total of 5454 differentially expressed genes (DEGs) and 41 differentially accumulated flavonoids were identified. Focusing on the flavonoid biosynthetic pathway, the analysis revealed that flavonoids, particularly myricetin, quercetin, and kaempferol derivatives, may play important roles in enhancing plant thermotolerance. Concurrently, specific genes within the *Rh4CL* and *RhFLS* families were significantly upregulated under high-temperature stress, suggesting their potential involvement in promoting flavonoid accumulation under heat stress. Furthermore, transcription factor analysis indicated that AP2/ERF-ERF, MYB, and NAC transcription factors may participate in the response to high-temperature stress and the regulation of flavonoid biosynthesis in *R. hainanense*. These findings provide important clues for elucidating the accumulation patterns of flavonoids under high-temperature stress and offer candidate genes for further research on flavonoid biosynthetic pathways.

## Data Availability

The original contributions presented in this study are included in the article/Supplementary Materials. Further inquiries can be directed to the corresponding author.

## Supplementary Materials

The supporting information can be downloaded at https://www.mdpi.com/article/10.3390/plants15060964/s1. Table S1. Classification of differentially expressed transcription factors.

## Abbreviations

The following abbreviations are used in this manuscript:

PAL	Phenylalanine ammonia-lyase
C4H	Cinnamate 4-hydroxylase
4CL	4-coumarate:CoA ligase
CHS	Chalcone synthase
CHI	Chalcone isomerase
F3H	Flavanone 3-hydroxylase
FLS	Flavonol synthase
DFR	Dihydroflavonol 4-reductase
ROS	Reactive oxygen species
DAMs	Differentially accumulated metabolites
DEGs	Differentially expressed genes
